# A comprehensive overview of yeast libraries and their role in advancing cell biology

**DOI:** 10.1111/febs.70325

**Published:** 2025-11-21

**Authors:** Din Baruch, Maya Schuldiner, Ofir Klein

**Affiliations:** ^1^ Department of Molecular Genetics Weizmann Institute of Science Rehovot Israel

**Keywords:** functional genomics, gene deletion, high‐throughput screening, protein localization, protein tagging, protein–protein interactions, SWAp‐Tag (SWAT), Synthetic Genetic Array (SGA), yeast libraries

## Abstract

Over the past two decades, genome‐wide collections of mutants, or libraries, have revolutionized the fields of systems and cell biology by enabling systematic and high‐throughput interrogation of gene and protein function. This has been especially prominent in the model yeast *Saccharomyces cerevisiae*. The unique genetic properties of yeast, combined with efficient genome engineering tools, have facilitated the creation of a large number of comprehensive collections of strains with targeted gene deletions, mutations, overexpressions, regulatable promoters, and protein tagging. These resources have enabled large‐scale studies of cellular phenotypes, genetic and drug interactions, protein localization, protein–protein interactions, and much more. This review provides a comprehensive overview of the available systematic yeast libraries, highlighting their design, applications, and transformative impact on functional genomics. We detail how successive generations of libraries have addressed key challenges. Highlighting future applications, we discuss the potential integration of advanced fluorescent tools and machine learning approaches that promise to shape the next generation of libraries and establish yeast as a blueprint for systematic, dynamic, and predictive cell biology.

## Graphical abstract

Systematic, genome‐wide yeast mutant libraries have revolutionized systems and cell biology. By allowing high‐throughput interrogation of every gene and protein, they have defined gene essentiality, charted protein localization, illuminated function, mapped interactions, and more. This review provides a comprehensive overview of these resources, emphasizing their design, applications, and transformative role in functional genomics and discusses their future potential.

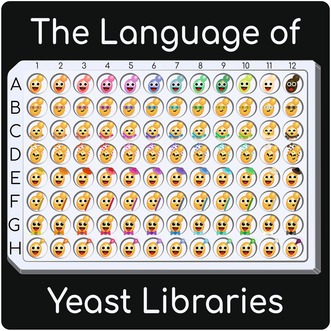

**Abbreviations**
ABOLISH: auxin‐inducible biotin ligase suppression system
AI: artificial intelligence
AID*: auxin‐inducible degron (optimized variant)
AVI tag: biotin acceptor peptide tag
BioID: proximity‐dependent biotin identification
C′/N′: C‐ or N‐terminal
DAmP: decreased abundance by mRNA perturbation
DHFR: dihydrofolate reductase
eGFP: enhanced green fluorescent protein
FP: fluorescent protein
GFP: green fluorescent protein
HA: hemagglutinin epitope tag
HRV: human rhinovirus (3C protease cleavage site)
LgBiT/SmBiT: large BiT/Small BiT fragments of NanoLuc luciferase
mNG: monomeric NeonGreen fluorescent protein
mScarlet‐I: monomeric scarlet‐I fluorescent protein
MTS: mitochondrial targeting signal
MTX: methotrexate
NanoBiT: NanoLuc Binary Technology (split‐luciferase system)
NanoLuc: engineered luciferase from *Oplophorus gracilirostris*
OD: optical density (at 600 nm, OD₆₀₀)
PCA: protein‐fragment complementation assay
PCR: polymerase chain reaction
PPI: protein–protein interaction
scFv: single‐chain variable fragment
sfGFP: superfolder green fluorescent Protein
SGA: Synthetic Genetic Array
SGD: Saccharomyces Genome Database
SP: signal peptide
SWAT: SWAp‐Tag
TAP: tandem affinity purification
TetO7: tetracycline‐repressible promoter
tFT: tandem fluorescent protein timer
ts: temperature‐sensitive (allele)
UTR: untranslated region
YETI: Yeast Estradiol Strains with Titratable Induction

## Introduction

The budding yeast *Saccharomyces cerevisiae* (yeast from now on) stands out as one of the most extensively studied eukaryotic model organisms due to its rapid growth, the availability of advanced genetic tools, and its genetic simplicity. It was also the first eukaryote to be fully sequenced, already three decades ago [1], revealing that it has fewer than 6000 protein‐coding genes [2]. Moreover, only ~5% of yeast genes contain introns, typically only a single one, with negligible alternative splicing [3], ensuring that almost every gene encodes a single protein. Notably, since 64% of yeast genes are conserved all the way to humans [4], research in yeast has transformed our understanding of cell biology and genetics in both systems, and provided the foundational gene orthology annotations that drive much of modern functional genomics. The properties of the yeast model system suggested a unique opportunity for a comprehensive analysis of its genome and proteome, leading to a surge in systems‐biology research and a unique opportunity to explore cell biology.

**Fig. 1:**
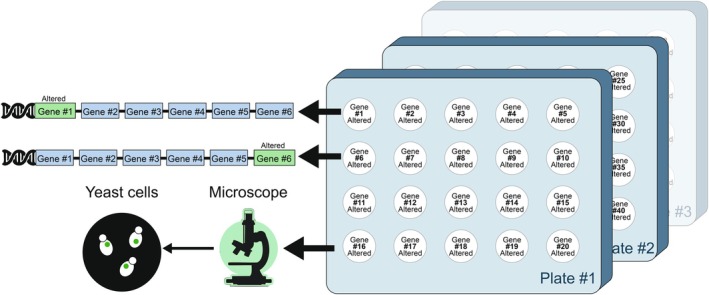
Schematic illustration of an arrayed yeast library. The schematic illustration represents a collection, or library, of genomically modified and arrayed yeast strains. Each spot on the plates represents a yeast colony, with each colony harboring a different genomic alteration, resulting in a modified version of a specific gene. The colonies are arrayed on solid agar plates or liquid multi‐well plates, enabling precise, convenient strain identification, maintenance, handling, tracking, and parallel analysis of thousands of strains. For example, as illustrated in the figure, fluorescently labeled strains can be imaged under an automated microscope, systematically revealing subcellular features in each strain.

The availability of the full‐genome sequence, the finite and small number of genes, and the creation of highly efficient PCR‐mediated homologous recombination techniques [5, 6, 7, 8, 9] brought about a new era where it became possible and feasible to develop systematic collections of genetically modified and arrayed yeast strains, colloquially known as yeast libraries. These libraries serve as organized resources where genes are systematically altered in a similar manner to alter gene expression or create changes in the proteins encoded by them (Fig. 1). These include gene deletion or mutation, promoter swapping, or the addition of a fusion sequence (‘tagging’), thus allowing the study of their roles in a systematic and high‐throughput manner. The significance of yeast libraries lies not only in their practical advantages, such as enabling faster, cheaper, and more standardized access to genetically modified strains, but also, and more profoundly, in the new types of experiments they made possible. Thanks to yeast libraries, for the first time, researchers could perform genome‐wide comparisons across all yeast genes in a single, systematic experiment. This shift enabled the collection of rich phenotypic data at an unprecedented scale and allowed direct comparisons across the entire genome. Such capabilities opened the door to extracting numerical measures of functional relationships and gene networks in a way that was previously unattainable. This paradigm has since inspired similar large‐scale efforts in other systems like bacteria [10, 11], chlamydomonas [12], drosophila [13], zebrafish [14], plants [15, 16, 17, 18, 19, 20, 21], and systems, such as the DepMap project in mammalian cells [22].

Before the construction of systematic libraries, genetic screens based on random mutagenesis were a groundbreaking approach that led to the discovery of fundamental cellular processes [23, 24]. Classic screens, such as those, helped to uncover key pathways in the cell cycle, DNA repair, autophagy, vesicle trafficking, and more. While such screens led to seminal discoveries (and several Nobel prizes), they were laborious to set up and follow up, needing to trace back the mutants which resulted in a phenotype. In contrast, libraries brought structure and precision, enabling researchers to interrogate every gene in a controlled and replicable manner. Indeed, this is clear from the ability of such studies to answer fundamental biological questions, as can be seen from the ~15 000 citations that reference yeast libraries (data retrieved from ‘Web of Science’, accessed April 2025). For a few examples (out of manuscripts and approaches that are too numerous to mention and cite): by assaying the growth rate of every single deletion strain under multiple conditions, it was possible to decipher which genes are essential for survival under specific conditions [25, 26, 27] or tracking their genetic interactions and measuring how they fare on various genetic backgrounds [28, 29]. Alternatively, libraries that represented each gene fused to a fluorescent protein allowed the determination of protein localizations under varying conditions [30, 31, 32, 33, 34, 35, 36, 37, 38]. Other libraries enable the monitoring of protein–protein interactions (PPI) systematically, creating a whole‐proteome network analysis [39, 40, 41].

Notably, yeast libraries are not isolated tools. Existing libraries can be genetically modulated to create ‘custom‐made’ collections systematically using the Synthetic Genetic Array (SGA) method [29, 42, 43]. SGA relies on high‐density pinning approaches and sophisticated genetic selection steps to automate large‐scale genetic manipulation of libraries. This enables the integration of any genetic alteration of choice (e.g., a fusion gene or a deletion background) into the background of an entire library by simply crossing it with a donor strain of interest. The flexibility to manipulate yeast libraries allows the creation of endless combinations to address specific questions. For example, it is possible to view the localization of one protein on the background of mutations in all others, uncovering targeting factors and regulators. Conversely, it is possible to assay the impact of a deletion in one gene on the abundance and localization of all proteins, thereby understanding its impact on cellular rewiring. The availability of multiple current methods for systematic phenotyping (such as microscopy, flow cytometry, mass spectrometry, and sequencing) allows assessment of phenotypes ranging from growth, through localization, proteomics/metabolomics/lipidomics, gene expression, and more. Furthermore, this flexibility also provides the potential to adapt yeast libraries for future methods, making yeast libraries an extremely powerful tool in the research of cellular physiology.

While some collections have been made using plasmids or have resulted in pools of strains, this review will focus only on the vast collection of arrayed yeast libraries where modifications were genomically integrated and revolved around protein‐coding genes. We have highlighted their design and touched upon their applications and contributions to biological research (whose full scope exceeds the limits of this review). To facilitate an overview, we provide comprehensive tables detailing the major yeast libraries and their unique features. This table is also updated regularly on the ‘LibrarYeast’ website (MayaSchuldiner.wixsite.com/SchuldinerLab/LibrarYeast) to enable it to stay current as more libraries are continuously being made.

Importantly, as systematic yeast libraries become more widely adopted, it is important to remain aware of their inherent limitations. For example, well‐to‐well contamination can occur during handling, and strains may accumulate additional mutations elsewhere in the genome. Such secondary mutations can act as suppressors that mask or compensate for the phenotype of the intended mutation. These issues can occasionally lead to false‐positive or false‐negative results. To address these challenges, the field has developed complementary libraries—collections that target the same set of genes but through independent strategies. Because each library perturbs gene function in a distinct manner, they provide orthogonal ways of testing the same biological question. Examples include the combination of complete gene deletion collections with conditional depletion systems, such as degron‐tagging or inducible promoters. This redundancy is a major strength: it enables researchers to cross‐validate phenotypes and obtain deeper biological insights. We therefore recommend validating individual strains through sequencing and PCR when appropriate, particularly in follow‐up experiments. By combining careful validation with the strategic use of complementary resources, the yeast community is well positioned to continue advancing functional and systems biology.

## Setting the cornerstone—first‐generation collections

The yeast knockout (YKO) library was one of the first large‐scale collections in which each individual gene was removed, allowing its function to be assayed [26, 27]. Importantly, this collection enabled pooled fitness assays by including molecular barcodes for each deletion strain. Since, at the time of construction of this collection, it was not clear which genes were essential, strains were first constructed as heterozygous diploid deletion strains, which were sporulated to assess gene essentiality. This approach identified 1105 essential genes in rich glucose medium. Furthermore, the genome‐wide deletion collection enabled the development of SGA analysis, which systematically characterizes genetic interactions by combining mutations in different genes to reveal functional relationships and networks [29]. The YKO collection and related technologies have been instrumental in advancing genome‐wide functional genomics, setting a foundation for subsequent genetic interaction mapping and complex trait analysis.

At the same time, complementary efforts were made to create libraries that will provide powerful tools for studying proteins directly, rather than genes. These include libraries to measure localization, abundance, and interactions. The C‐terminal (C′) green fluorescent protein (GFP)‐tagged library enabled live‐cell imaging under the regulation of each protein's native promoter [33]. The C′ GFP library allowed the first assignment of localizations to the majority of the proteome, including many proteins that were never studied before. Complementing this, the tandem affinity purification (TAP) tag library created C′ fusions with a tag that is designed for protein purification and detection, which again preserved regulation by the native promoter, thus facilitating the measurement of protein abundance [44, 45, 46]. Using this collection, it was demonstrated that 80% of the proteome is expressed under standard growth conditions (rich medium, 30 °C, mid‐log phase), with protein levels spanning a wide dynamic range of fewer than 50 to over 1 000 000 molecules per cell. The versatility of the TAP tag also allowed for further genome‐wide measurements of protein half‐life [47], as well as protein complex purification and functional network mapping [39, 40, 48].

These resources resulted in a rapid accumulation of systematic data, and their usefulness was augmented by the powerful and efficient curation into the *Saccharomyces* Genome Database (SGD) [3]. In addition, to enable access to the data from the original C′ GFP and TAP library papers, a searchable website was created [49]. Later, a database tracking visualization of the GFP library under multiple conditions was also created [30, 50]. These databases also benchmarked the importance of the sharing of data by the yeast community, which continues to this day.

These early yeast libraries transformed genetic and proteomic studies, shifting from single‐gene analyses to comprehensive, genome/proteome‐wide approaches. They laid the foundation for the next generation of yeast libraries by standardizing gene deletion, tagging, large‐scale screening, and open data access. For further details of the libraries in this section, see Table 1.

**Table 1:** First‐generation collections.

Library	Description	Location of tag	Mating type	Genotype	References	Coverage
Deletion	A collection where each gene is precisely deleted and replaced with a kanMX4 (encoding G418 Resistance) selection marker. This deletion strategy combines molecular barcodes (UPTAG and DNTAG) flanking the marker, enabling high‐throughput identification and quantification of each strain even in pooled cultures. The collection includes haploid (both mating types) and diploid versions, allowing for the assessment of both essential (as heterozygous diploids) and nonessential genes	NA	MATa MATα Diploid	(a) *his3∆1 leu2∆0 met15∆0 ura3∆0 **∆xxx::UPTAG‐G418R‐DNTAG*** (α) *his3∆1 leu2∆0 lys2Δ0 ura3∆0 **∆xxx::UPTAG‐G418R‐DNTAG*** (a/α) *his3∆1/his3∆1 leu2∆0/leu2∆0 met15∆0/MET15 LYS2/lys2Δ0 ura3∆0/ura3∆0 **∆xxx::UPTAG‐G418R‐DNTAG/XXX***	[26, 27]	Full genome (5120 genes)
C′ GFP	Each gene is altered such that the protein it encodes is fused at the C terminus with a green fluorescent protein (GFP)	C′	MATa	*his3Δ1 leu2Δ0 met15Δ0 ura3Δ0 **XXX‐GFP‐HIS3***	[33]	Full genome (5206 genes)
C′ TAP tag	Each gene is altered such that the protein it encodes is fused at the C terminus with a tandem affinity purification (TAP) tag. This tag allows immunodetection using a single antibody, while purification and interactome analyses are typically performed by sequential binding to IgG and calmodulin beads	C′	MATa	*his3∆1 leu2∆0 met15∆0 ura3∆0 **XXX‐TAP‐HIS3***	[44]	Full genome (4715 genes)

The text that is not in bold indicates the genetic background of the library, and the text in bold indicates the genetic modification that was made to each and every gene in the library.

## Filling the gap—essential and small genes and strain diversification

A major gap in the first libraries was that small genes were not covered. This is because the first annotation of the yeast genome only considered genes encoding proteins of over 100 amino acids [2]. Upon the annotation of small genes, an additional library was developed to cover their deletions [51], in the same genetic background as the original deletion library and with the same selection cassette and molecular barcoding system. Therefore, users relying on the deletion library can incorporate this collection to expand coverage. For researchers using other types of libraries, which do not cover the small genes (such as the C′ GFP and TAP libraries), it is important to be aware that they are not represented, which can potentially lead to incomplete interpretations.

While the deletion library provided invaluable insights into the phenotypes of nonessential genes, it had limited ability to study the function of essential ones, which could only be studied in heterozygous diploids [26, 27]. To enable systematic analysis of essential genes, several approaches for systematically generating reduced or conditional expression or function were explored.

The earliest approach leveraged transcriptional modulation. In this approach, promoters of essential genes were swapped with an inducible or repressible promoter. Namely, the tetracycline‐repressible promoter (TetO7) library [52] introduced a TetO7 promoter upstream of the coding sequence of approximately two‐thirds of essential genes, enabling conditional and reversible shut‐off of gene expression upon the addition of doxycycline. This tetracycline analog inhibits transcription by interfering with a regulatory transactivator bound to the TetO7 promoter. Using doxycycline, extensive gene depletion, resulting in severe growth defects or lethality, was demonstrated for nearly 500 strains from the collection. An additional collection, the Yeast Estradiol Strains with Titratable Induction (YETI) library [53], was more recently made with the approach of exchanging the endogenous promoter for a synthetic one. This library employs an estradiol‐responsive transcriptional activator (Z3EV system) to regulate genes using an inducible promoter activated by β‐estradiol [54]. The YETI collection is genome‐wide and not just for essential genes; thus, it enables genome‐wide tunable and reversible modulation of protein levels. Moreover, unlike most other libraries made in the BY4741 background, the YETI library was made in the prototrophic RCY1972 strain, expanding the range of strains and conditions that can be tested.

An additional approach utilized Temperature‐Sensitive (ts) alleles, which retain gene function at a permissive temperature (25 °C) and disrupt it at a restrictive temperature (32–37 °C, strain dependent). Two libraries were created using this approach. The first ts library was constructed through a diploid shuffle approach [55, 56, 57], leaving the natural promoter intact. This library also included molecular barcodes for strain recognition in pools. Since the first library covered only about a quarter of essential genes, a complementary ts library was created [58]. The second library added hundreds of ts alleles, with many genes represented by multiple independent alleles. A large subset of strains is also molecularly barcoded.

A third approach was taken by the Decreased Abundance by mRNA Perturbation (DAmP) hypomorphic allele library [59, 60]. By replacing endogenous 3′ untranslated regions (UTRs) with an antibiotic resistance cassette (KanMX). This method led to a 2‐ to 10‐fold reduction in transcript levels. This approach complements promoter changes or ts alleles by affecting transcript stability rather than the complete elimination of the protein or interference with its sequence.

When genes are perturbed constitutively, such as by deletions or DAmP approaches, yeast cells can undergo compensatory adaptations, including the emergence of second‐site suppressors or broader transcriptional rewiring [61, 62, 63]. This adaptation can obscure the immediate, primary phenotype of gene loss and complicate phenotype interpretation. To address this, a set of libraries enabling the study of protein loss ‘on demand’ and prior to such rewiring, utilizing the auxin‐inducible degron (AID) method, was developed [64, 65]. These libraries are based on a rapid, reversible protein depletion method driven by the AID* system through the TIR1 ubiquitin‐proteasome adaptor that is activated upon 5‐Ph‐IAA (an auxin analog) addition [66, 67]. These libraries contain strains with C′ AID* in fusion, with some also encoding an additional detectable tag. The versions with enhanced GFP (eGFP) or monomeric NeonGreen (mNG) fusions enable fluorescent tracking of protein depletion *in vivo*, and the version with 3myc tag allows tracking of degradation by immunodetection. In the AID*‐eGFP collection, there is a constitutive expression of OsTIR1_(F74G)_ [65], whereas the mNG‐AID* library harbors the TIR1 under the control of the inducible *GAL1* promoter [64], enabling dual control of degradation timing. To enable easy access for the yeast community to the phenotypic outcome of these protein depletions, a database was created [65].

Finally, most libraries mentioned until now were made with strains derived from the same genetic background—S288C [68]. While these strains have become predominant as a cell biology model, they lack certain aspects of natural yeast biology. One such trait is filamentous growth, which does not occur in S288C. To complement this, the Sigma yeast deletion collection [69] was created. This collection mirrors the original deletion library design but was constructed in the filamentation‐competent Ʃ1278b strain background. In this collection, genes involved in morphogenetic switching and pseudohyphal development can be systematically interrogated. The Sigma collection includes both heterozygous and homozygous diploid deletions and has been instrumental in identifying genetic regulators of invasive growth, biofilm formation, and pseudohyphal development. Since gene essentiality can differ between strain backgrounds, this collection also provides complementary insight into essentiality and phenotype that may be obscured in S288C‐based libraries.

It is highly beneficial for the comprehensive exploration and understanding of essential proteins to have multiple complementary libraries, as each has its own limitations but also provides unique capabilities [70]. For example, ts libraries require elevated temperatures for phenotyping, which may introduce unintended secondary effects. Promoter‐swap libraries allow conditional expression or overexpression, both of which potentially lead to cellular rewiring over time. Additionally, they can suffer from leakiness or imprecise control over protein levels, and the synthetic drugs used could cause cellular side effects. Degradation‐based libraries rely on targeted protein degradation, yet some proteins remain physically or structurally unreachable to the degradation machinery and thus inaccessible for these studies. The DAmP approach is highly variable and does not result in the complete elimination of proteins, allowing residual protein activity that may complicate the discovery of the underlying phenotypes of functional loss. Since no single method is universally optimal, selecting the most appropriate library based on specific experimental needs is needed. Often, integrating multiple approaches to overcome individual limitations and gain a more complete functional characterization of essential proteins is the best choice.

For further details on the libraries in this section, see Table 2.

**Table 2:** Essential and small genes and strain diversification.

Library	Description	Location of tag	Mating type	Genotype	References	Coverage
Mini ORFs	Deletions of small open reading frames (sORFs, <100 amino acids), completing the deletion collection. Including molecular barcodes like those in the yeast deletion library. Built with the same selection markers and the same genotype as the yeast deletion library so that they can be combined.	NA	MATa MATα Diploid	(a) *his3∆1 leu2∆0 met15∆0 ura3∆0 **∆xxx::G418R*** (α) *his3∆1 leu2∆0 lys2Δ0 ura3∆0 **∆xxx::G418R*** (a/α) *his3∆1/his3∆1 leu2∆0/leu2∆0 met15∆0/MET15 LYS2/lys2Δ0 ura3∆0/ura3∆0 **∆xxx::G418R/XXX***	[51]	247 small genes
TEToff promoter	The promoter of each essential gene is replaced with the tetracycline‐repressible promoter (TetO7). This design allows for conditional repression of essential genes by the addition of doxycycline	NA	MATa	*ura3∆0::URA3‐CMV‐tTA his3∆1 leu2∆0 met15∆0 **G418R‐TetO7pr‐XXX***	[52]	Essentials (892 genes)
YETI	The promoter of each gene is replaced with the β‐estradiol‐inducible Z3EV promoter. This system enables titratable gene induction/repression. The collection covers essential genes in diploid form (YETI‐E) and nonessential genes in haploid form (YETI‐NE). Each strain is also barcoded	NA	MATa Diploid	(a) ***barcode‐URA3‐Z3EVpr‐XXX** hap1∆::NATR‐ACT1pr‐Z3EVTF‐ENO2term ura3∆0 can1∆::STE2pr‐spHIS5 his3∆1 lyp1∆* (a/α) ***barcode‐URA3‐Z3EVpr‐XXX** hap1∆::NATR‐ACT1pr‐Z3EVTF‐ENO2term/HAP1 ura3∆0/ura3∆0 can1∆::STE2pr‐spHIS5/CAN1 his3∆1/his3∆1 lyp1∆/LYP1*	[53]	Genome‐wide
Temperature‐sensitive (ts) 2008	Essential Genes are represented by a temperature‐sensitive (ts) allele. These ts alleles allow normal protein function at a permissive temperature (25 °C) and impair function at a nonpermissive temperature (32–37 °C), enabling conditional analysis of essential genes. Each allele is flanked by UPTAG and DNTAG barcodes, enabling high‐throughput identification and quantification of each strain even in pooled cultures	NA	MATa	*ura3Δ0 leu2Δ0 his3Δ1 lys2Δ0 (or LYS2) met15Δ0 (or MET15)* *can1Δ::LEU2‐MFA1pr‐His3 **UPTAG‐XXX^ts‐URA3‐DNTAG***	[56, 57]	Essentials (362 genes)
Temperature‐sensitive (ts) 2011	This collection complements the 2008 ts library by adding strains carrying at least one conditional ts allele	NA	MATa	*his3∆1 leu2∆0 met15∆0 ura3∆0 **XXX^ts‐G418R***	[58]	Essentials (497 genes)
DAmP Hypomorphic Alleles	The 3′ untranslated region (UTR) of each essential gene is deleted, altering mRNA stability, causing reduced abundance (two to 10‐fold). Built with the same selection markers and genotype as the yeast deletion library, they can be combined	NA	MATa Diploid	(a) *his3∆1 leu2∆0 met15∆0 ura3∆0 **XXX‐DAmP‐G418R*** (a/α) *his3∆1/his3∆1 leu2∆0/leu2∆0 met15∆0/met15∆0 ura3∆0/ura3∆0 **XXX‐DAmP‐G418R/XXX***	[60]	Essentials (842 genes)
C′ AID‐eGFP	Based on the C′‐SWAT parental collection (see section on Modular innovation), each gene is altered such that the protein it encodes is tagged at the C terminus with a minimized auxin‐inducible degron (AID*) followed by an enhanced GFP (eGFP). This design allows both visualization of protein localization and abundance (through the GFP tag) and rapid, conditional protein depletion (using the AID system). Each strain also contains the modified OsTIR1(F74G). adaptor for E3 ubiquitin ligases. Upon the addition of the modified auxin analog 5‐Ph‐IAA, the Tir1 adaptor targets the AID* fused protein for degradation via the ubiquitin‐proteasome pathway	C′	MATα	his3∆1 leu2∆0 met15∆0 ura3∆0 can1∆::GAL1pr‐SceI‐STE2pr‐spHIS5 lyp1∆::STE3pr‐LEU2, **NATR‐TEF2pr‐OsTIR1(F74G)**, XXX‐AID*‐eGFP‐G418R	[65]	Genome‐wide
C′ AID‐Monomeric Neon Green (mNG)	Two libraries, all Based on the C′‐SWAT parental collection (see section on Modular innovation), and on AID*. V1: Each gene is altered such that the protein it encodes is tagged with mNG‐AID*‐3myc and is represented as either containing or lacking the OsTIR1 for control. V2: Each gene is altered such that the protein it encodes is fused to AID*‐3myc, and the OsTIR is regulated by the galactose inducible/glucose inhibited GAL1pr. These libraries offer versatile tools for conditional, proteome‐wide protein depletion	C′	MATα	V1 (OsTIR1‐ set): lyp1Δ his3Δ1 leu2Δ0 ura3Δ0 met15Δ0 can1Δ::STE3pr‐LEU2‐GAL1pr‐NLS‐I‐SCEI XXX‐mNG‐AID*‐3myc‐HygroR V1 (OsTIR1+ set): his3Δ1 leu2Δ0 ura3Δ0 met15Δ0 can1Δ::STE3pr‐LEU2‐GAL1pr‐NLS‐I‐SCEI lyp1Δ::GAL1pr‐OsTIR1(F74G)‐NATR XXX‐mNG‐AID*‐3myc‐HygroR V2 (OsTIR1+ set): his3Δ1 leu2Δ0 ura3Δ0 met15Δ0 can1Δ::STE3pr‐LEU2‐GAL1pr‐NLS‐I‐SCEI lyp1Δ::GAL1pr‐OsTIR1(F74G)‐NATR XXX‐AID*‐3myc‐HygroR	[64]	Genome‐wide
Sigma collection—Filamentous growth deletion	Gene deletions were introduced into the filamentation‐competent ∑1278b yeast strain background. The library includes both haploid and homozygous diploid deletions	NA	MATa MATα Diploid	(a) *can1Δ::STE2pr‐Sp_his5 lyp1Δ::STE3pr‐LEU2 his3::his3G leu2Δ ura3Δ **∆xxx::G418R*** (α) *can1Δ::STE2pr‐Sp_his5 lyp1Δ::STE3pr‐LEU2 his3::his3G leu2Δ ura3Δ **∆xxx::G418R*** (a/α) *can1Δ::STE2pr‐Sp_his5/CAN1 lyp1Δ::STE3pr‐LEU2/LYP1 his3::his3G/his3::his3G leu2Δ/leu2Δ ura3Δ/ura3Δ **∆xxx::G418R/∆xxx::G418R***	[69]	Full genome (4028 genes in haploids and 3900 genes in homozygous diploid)

The text that is not in bold indicates the genetic background of the library, and the text in bold indicates the genetic modification that was made to each and every gene in the library.

## Wiring the cell—Protein–protein interactions and dynamics

Functional genomics using gene mutations can reveal phenotypes under diverse conditions and provide a comprehensive view of genetic interactions [28, 29, 43, 71]. To complement these efforts, physical PPI assays are highly useful. The first generation of a yeast library that enabled this was the TAP‐tag library, which used protein purification and mass spectrometry (MS) [39, 40]. However, pull‐downs followed by MS are laborious methods, and so a new generation of libraries brought about alternative approaches for assaying PPIs that are also applicable *in vivo*. The first was a set of two dihydrofolate reductase (DHFR) protein‐fragment complementation assay (PCA) libraries [41]. This approach utilizes two complementary fragments of mutant murine DHFR, resistant to methotrexate (MTX). Each library has one of the DHFR parts fused to the C′ of each gene in the collection, in the opposing mating type. Since native yeast DHFR is essential for yeast viability and is completely inhibited by MTX, the addition of MTX to yeast is lethal. Based on this, mating strains from the two libraries allow the PPI between the fused proteins in the resulting diploid to be assayed. Specifically, interaction between the two tagged query proteins brings the murine DHFR fragments into proximity, reconstituting enzymatic activity and resistance to MTX, allowing growth on selective media as a sensitive readout. The DHFR PCA enabled genome‐wide mapping of 2770 interactions among 1124 proteins.

A complementary set of libraries for the discovery of PPIs is the split‐Venus libraries, which allow visualization of PPIs occurring *in vivo*, revealing their intracellular localization. In the split‐Venus libraries, proteins are C′‐tagged with either the N′ [72] or the C′ [73] portions of a split‐Venus fluorophore. Mating of strains from the two libraries allows assaying the PPI between the two fused proteins in the resulting diploid. Specifically, it enables reconstitution of the fluorophore if the two fusion proteins come within ~7–10 nanometers, allowing visualization of interactions in live cells. While this system is highly sensitive, as with all PCAs, signal complementation may result from spatial proximity rather than direct physical binding between proteins. Thus, positive signals can reflect either true binary interactions or co‐localization within the same complex or subcellular structure, depending on the flexible linker length and geometry and tag size [41, 74].

Many yeast libraries are most suited for static measurements, such as localization, abundance, or interaction. However, understanding protein turnover, inheritance, and segregation requires mapping protein dynamics. The tandem fluorescent protein timer (tFT) library [75, 76] was created to address this challenge. In this collection, proteins were seamlessly C′‐tagged with a dual fluorescent module: fast‐maturing superfolder GFP (sfGFP) and slow‐maturing mCherry. The resulting mCherry/sfGFP fluorescence ratio, which provides a quantitative readout of protein half‐life, enabled systematic, *in vivo* monitoring of protein degradation kinetics.

Put together, these collections enabled querying of protein traits and brought forward a better understanding of protein dynamics, function, and networks.

For further details on the libraries in this section, see Table 3.

**Table 3:** Libraries for protein–protein interactions and dynamics.

Library	Description	Location of tag	Mating type	Genotype	References	Coverage
C′ DHFR PCA	Two collections in which each gene is altered such that the protein it encodes is fused to complementary fragments of the methotrexate (MTX)‐resistant dihydrofolate reductase (DHFR), one part in each mating type, and with different selections. Upon mating of strains from the complementing libraries and diploid selection, if proteins interact, their fused DHFR fragments would also reconstitute enzymatic activity. Upon addition of MTX, the endogenous, essential DHFR is inhibited, allowing strains to grow only if the reconstituted DHFR is active	C′	MATa MATα	(a) *his3∆1 leu2∆0 met15∆0 ura3∆0 **XXX‐DHFR1,2‐NATR*** (α) *his3∆1 leu2∆0 lys2Δ0 ura3∆0 **XXX‐DHFR3‐HygroR***	[41]	Full genome (4320 genes in MATa and 4470 genes in MATα)
C′ split venus	Derived from the TAP library by manual replacement of the TAP cassette, each gene is altered such that the protein it encodes is C‐terminally tagged with split fragments of the Venus fluorescent protein: VN (N‐terminal part) and VC (C‐terminal part)	C′	MATa MATα	(a) *his3∆1 leu2∆0 met15∆0 ura3∆0 **XXX‐VN‐URA3*** (α) *his3∆1 leu2∆0 met15∆0 ura3∆0 **XXX‐VC‐LEU2***	[72, 73]	Full genome (5911 genes in MATa and 5671 genes in MATα)
Tandem fluorescent protein timer (tFT)	Each gene is altered such that the protein it encodes is seamlessly C‐terminally tagged with an immature tandem fluorescent timer (mCherry–SceI site‐URA3‐sfGFP). This form of the cassette allows for genetic crossing of the library with a strain of choice. Upon excision induction by galactose, the tandem fluorescent timer recombines to its mature form (mCherry–sfGFP), suitable for measuring protein age by a simple fluorescent readout	C′	MATα	Genotype before excision: *his3∆1 met15∆0* *ura3∆0 can1∆::STE2pr‐spHIS5 lyp1∆::STE3pr‐LEU2 leu2∆::GAL1pr‐I‐SCEI‐natNT2 **ORF‐mCherry‐SceI site‐SpCYC1term‐ScURA3‐SceI site‐mCherry∆N‐sfGFP***	[75]	Full genome (4044 genes)
				Genotype after excision: *his3∆1 met15∆0* *ura3∆0 can1∆::STE2pr‐spHIS5 lyp1∆::STE3pr‐LEU2 leu2∆::GAL1pr‐I‐SCEI‐natNT2 **ORF‐mCherry‐ ‐sfGFP***		

The text that is not in bold indicates the genetic background of the library, and the text in bold indicates the genetic modification that was made to each and every gene in the library.

## Modular innovation—SWAp‐tag (SWAT) strategy and its impact on yeast library development

**Fig. 2:**
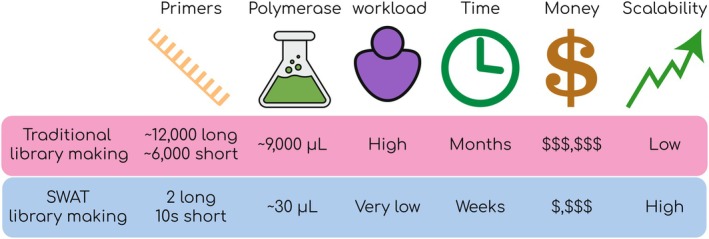
Comparison between the construction process of traditional and SWAT libraries. The figure compares key parameters involved in traditional genome‐wide yeast library generation versus the SWAT (SWAp‐Tag) method process. Parameters include the number of primers required, polymerase volume, workload, time, cost, and scalability. Traditional methods demand thousands of primers and high reagent volumes, resulting in significant workload, time investment (months), and cost, with limited scalability. In contrast, the SWAT approach requires only a few primers and minimal polymerase, dramatically reducing workload, time (weeks), and cost while offering high scalability.

Traditional genome‐wide yeast libraries (discussed above) were generated through labor‐intensive PCR‐mediated homologous recombination techniques, where each gene was individually modified in a common genetic background, one strain at a time. Each modification required new transformation, selection, and verification processes, making large‐scale library production cumbersome, time‐consuming, and costly (Fig. 2). Therefore, few laboratories could make a yeast library, so only a few full‐genome collections were made.

To overcome these challenges, the SWAp‐Tag (SWAT) strategy [77] introduced a modular approach for genomic tagging, enabling rapid and highly efficient integration of genetic cassettes directly into desired endogenous chromosomal loci through *in vivo* recombination. This method utilized a universal acceptor module inserted either upstream [38, 77] or downstream [36] of genes, serving as a placeholder that could be efficiently replaced. In short, a new SWAT library can easily be made by mating a parental library (N′ or C′) with a donor strain harboring the SGA markers, an SceI restriction enzyme under a *GAL1*pr, and a donor plasmid containing the new desired genetic modification. The library is then subjected to SGA methodology, followed by plating on galactose‐containing media to induce expression of the SceI endonuclease. SceI introduces double‐strand breaks only at its recognition sites, a long, 18‐base pair sequence that is absent from the native yeast nuclear genome and is present at both the acceptor and donor cassettes. The resulting breaks stimulate homologous recombination between matching linker sequences in the cassette and the plasmid, enabling efficient, *in vivo* exchange of sequences and creating a new library. Original, unswapped strains are selected against using 5FOA counter‐selection for the *URA3* marker in the parental placeholder.

**Fig. 3:**
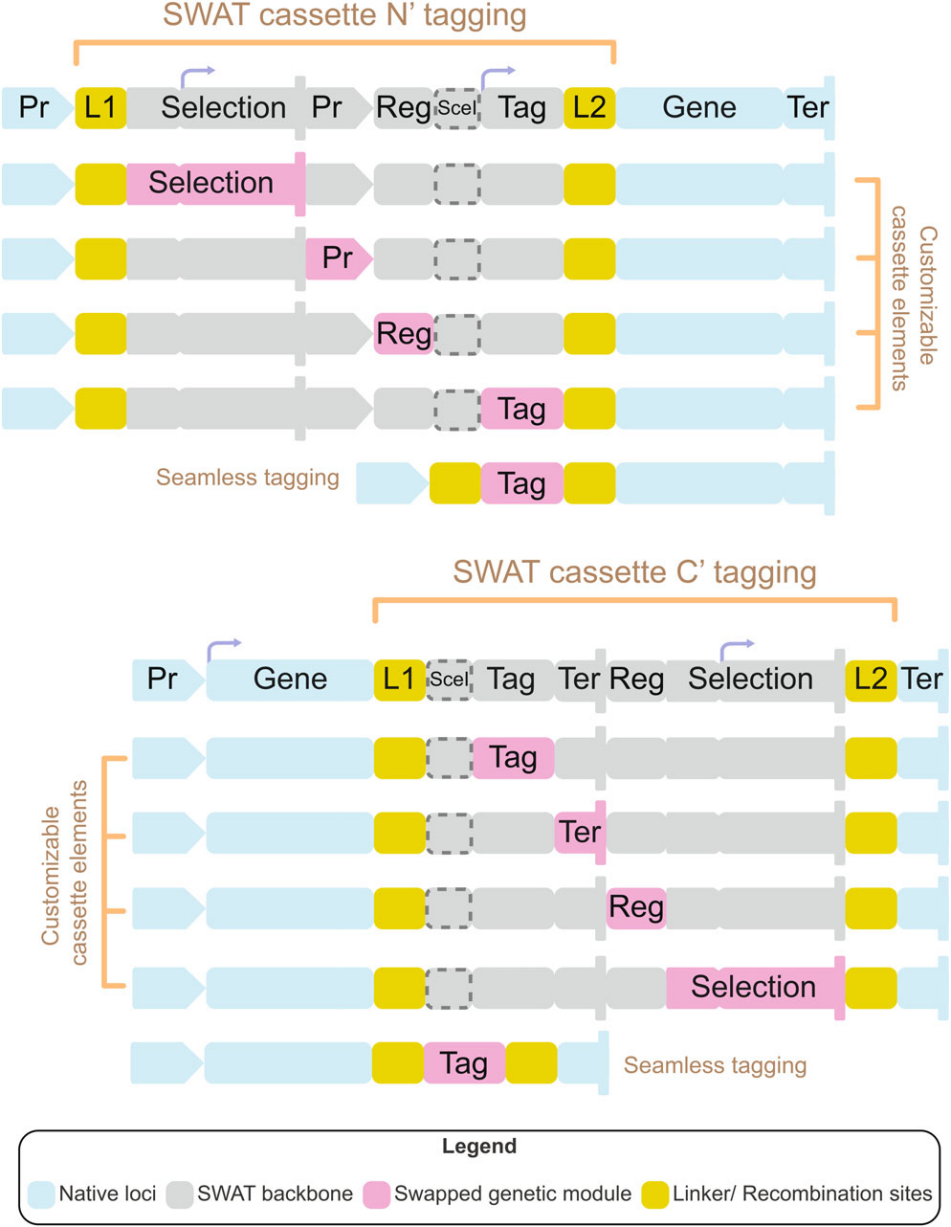
Modular design of SWAT cassettes enables genetic customization. An illustration of the structure and flexibility of the SWAT (SWAp‐Tag) system for both the N‐terminal (top) and C‐terminal (bottom) collections. Each SWAT cassette is flanked by recombination/linker sites (L1 and L2, yellow) and consists of a cassette (gray) composed of different genetic elements (magenta), such as selection markers, promoters (Pr), regulatory elements (Reg), tags, or terminators (Ter), any of which can be exchanged for alternative elements of choice or eliminated (such as in the case of seamless tagging). The cassette also contains SceI restriction site which is used to induce omologous recombination during the SWAT procedure. The modularity of the SWAT platform supports rapid, efficient, cost‐effective, and systematic generation of diverse genomic configurations, empowering large‐scale functional studies.

The modular SWAT design enables the seamless exchange of any genetic component, such as tag (fluorescent markers, degrons, affinity tags, etc.), promoter or terminator regions, UTRs, selection cassettes, or the insertion of any other sequence before or after the gene (Fig. 3). Indeed, the modularity of SWAT greatly enhanced the adaptability of yeast libraries, making it possible for any laboratory to customize strain collections for specific experimental needs without spending large budgets or years of time. The post‐SWAT era enabled an explosion of new collections to fit various experimental needs and to answer different biological questions.

For further details on these libraries, see Table 4.

**Table 4:** SWAT mother libraries.

Library	Description	Location of tag	Mating type	Genotype	References	Coverage
N′‐SWAT parental	Each gene is altered such that the protein it encodes is N‐terminally tagged with GFP and expressed under the constitutive NOP1 promoter, flanked by the SWAp‐Tag (SWAT) sequences to enable cassette swapping. To ensure proper targeting, proteins containing a Mitochondrial Targeting Signal (MTS) or a Signal Peptide (SP) also have a synthetic MTS/SP before the GFP tag, respectively	N′	MATa	*his3∆1 leu2∆0 met15∆0 ura3∆0* ***Hygro∆N′‐URA3‐spNOP1pr‐sfGFP‐XXX***	[38, 77]	Full genome (5457 genes)
C′‐SWAT parental	Each gene is capped by a CYC1 terminator flanked by the SWAT sequences to enable high‐throughput modification of the 3′ of the gene	NA	MATa	*his3∆1 leu2∆0 met15∆0 ura3∆0* ***XXX‐CYC1term‐scURA3‐HygroRΔN'‐ALG9term***	[36]	Full genome (5661 genes)

The text that is not in bold indicates the genetic background of the library, and the text in bold indicates the genetic modification that was made to each and every gene in the library.

## A new lens on the proteome—SWAT protein localization collections

The N‐terminal (N′)‐SWAT parental library [38, 77] itself already harbors a sfGFP fusion cassette under the *NOP1* constitutive promoter. This allowed, for the first time, imaging the localization of the proteome when tagged at the N′. Notably, whereas C′ tagged libraries interfere with important C′ sequences, such as peroxisomal targeting sequences, secretory pathway retrieval sequences, tail‐anchors, GPI anchoring sequences, lipidation sites, and the endogenous 3′ UTRs, which can contain important regulatory elements, genes harboring N′ tagging suffer from interference with the endogenous promoter and 5′ UTRs (which may affect translational regulation) as well as N′ targeting signals, such as mitochondrial targeting sequences (MTS) or signal peptides (SP). To minimize the interference with N′‐targeting signals, the parental N′‐SWAT library was built so that proteins with either MTSs or SPs are fused to a fluorophore preceded by a synthetic targeting signal to maintain protein targeting. This library provided systematic protein localization data, identifying locals for 796 proteins that could not be accurately visualized with C′ tagging. Interestingly, 636 proteins displayed different localization patterns between N′ and C′ tagging, revealing potential dual localization or termini preference for tagging [78].

In addition, since N′‐tagging interferes with natural promoter and 5′ UTR sequences, a NATIVEpr‐sfGFP library was made through the SWAT procedure [38, 77], resulting in a library where each protein is N′‐tagged with sfGFP, downstream of its native promoter, 5′ UTR and, if relevant, MTS or SP. The NATIVEpr‐sfGFP library identified conditionally expressed proteins and provided insights into the differences between constitutive and native promoter‐driven effects on expression and localization.

Expanding on the N′‐SWAT platform, the SWAT *TEF2*pr‐mCherry library [38, 77] introduced a red fluorescent‐tagged overexpression system using the strong *TEF2* promoter. This resulted in proteins tagged at the N′ with mCherry, ensuring high and constitutive expression. This library was particularly useful for co‐localization experiments, as it could easily be mated to existing GFP collections, and in the resulting diploids, the red fluorescently tagged protein could be imaged alongside GFP‐tagged ones to assess whether two proteins occupy the same subcellular compartment *in vivo*.

In the C′‐SWAT library [36], the downstream acceptor module positioned immediately before the stop codon of the respective gene allows the creation of C′ fusions. Using this platform, the C′‐SWAT mNG library incorporates this highly photostable and bright fluorescent tag, enabling high‐sensitivity imaging and quantitative protein expression analysis without disrupting N′ targeting or endogenous promoter regulation, although it does involve the replacement of the endogenous terminator with the *ADH1* terminator [36]. Importantly, the study also introduced a seamless tagging library and quantitatively compared the strength of various terminators relative to the generic ADH1 terminator, enhancing the understanding of how terminator choice affects protein expression. In addition, the C′‐SWAT platform was used to generate a proteome‐wide mScarlet‐I library, a red fluorescent protein with superior brightness and photostability, expanding the available palette of fluorescent reporters for dual‐color imaging and multiplexed localization studies [36]. Following in the footsteps of data sharing with the community, the associated resource, YeastRGB, offers an online database for visualizing and exploring the C′ GFP collection alongside the N′ SWAT NOP1pr‐GFP and C′‐SWAT mNG libraries [32].

For further details on these libraries, see Table 5.

**Table 5:** Fluorescent SWAT libraries for protein localization studies.

Library	Description	Location of tag	Mating type	Genotype	References	Coverage
N′ SWAT NATIVEpr‐GFP	Derived from the parental N′‐SWAT collection, each gene is altered such that the protein it encodes is N‐terminally tagged with GFP without altering the native sequence of the promoter and N‐terminal targeting signals (MTS or SP)	N′	MATα	*his3∆1 leu2∆0 met15∆0 ura3∆0* *can1∆::GAL1pr‐SceI‐STE2pr‐spHIS5 lyp1∆::STE3pr‐LEU2* ***XXXpr‐sfGFP‐XXX***	[38, 77]	Genome‐wide
N′ SWAT TEF2pr‐mCherry	Derived from the parental N′‐SWAT collection, each gene is altered such that the protein it encodes is N‐terminally tagged with mCherry and expressed under the strong, constitutive TEF2 promoter	N′	MATα	*his3∆1 leu2∆0 met15∆0 ura3∆0* *can1∆::GAL1pr‐SceI‐STE2pr‐spHIS5 lyp1∆::STE3pr‐LEU2* ***NATR‐TEF2pr‐mCherry‐XXX***	[38, 77]	Genome‐wide
C′‐SWAT mNG	Based on the C′‐SWAT parental collection, each gene is altered such that the protein it encodes is tagged at the C terminus with the bright fluorescent protein mNG followed by ADH1 terminator	C′	MATa	*his3∆1 met15∆0 ura3∆0* *leu2∆0::GAL1pr‐NLS‐SceI‐NATR can1∆::STE2pr‐spHIS5 lyp1∆::STE3pr‐LEU2* ***XXX‐mNG‐ADH1term‐HygroR***	[36]	Genome‐wide
C′‐SWAT mNG‐NATIVEterm	Based on the C′‐SWAT parental collection, each gene is altered such that the protein it encodes is tagged at the C terminus with the bright fluorescent protein mNG without altering the native sequence of the terminator	C′	MATa	*his3∆1 met15∆0 ura3∆0* *leu2∆0::GAL1pr‐NLS‐SceI‐NATR can1∆::STE2pr‐spHIS5 lyp1∆::STE3pr‐LEU2* ***XXX‐mNG ‐NATIVEterm***	[36]	Genome‐wide
C′‐SWAT mScarlet‐I	Based on the C′‐SWAT parental collection, each gene is altered such that the protein it encodes is tagged at the C terminus with the bright fluorescent protein mScarlet‐I	C′	MATa	*his3∆1 met15∆0 ura3∆0* *leu2∆0::GAL1pr‐NLS‐SceI‐NATR can1∆::STE2pr‐spHIS5 lyp1∆::STE3pr‐LEU2* ***XXX‐mScarlet‐I‐ADH1term‐HygroR***	[36]	Genome‐wide

The text that is not in bold indicates the genetic background of the library, and the text in bold indicates the genetic modification that was made to each and every gene in the library.

## Expanding the interaction landscape—SWAT PPI Libraries

To expand on the existing C′‐tagged PCA libraries, the same approaches were also established based on the N′ library, resulting in a broader coverage of the interactome. For example, a split‐DHFR system [38] was created such that SWATting was used to create two libraries in opposite mating types, where in each one the genes encode for proteins N′ fused to one of the two DHFR fragments. These strains employed seamless tagging, which does not have a selection attached to the genetic alteration. Hence, to allow for mating and selection of diploids of all protein–protein combinations, a selection marker was introduced in a distal site. The new DHFR libraries were made so that they can be assayed combinatorially [38] with the existing C′ DHFR library.

An additional approach used was the split Venus [38]. Similarly, two SWAT procedures allowed the creation of two libraries in opposite mating types, where in each one the genes encode proteins fused to one of the two Venus fragments (VN or VC). In addition to mating to each other to assay PPIs, it is possible to mate the split‐Venus libraries with a strain expressing a complementing cytosolic fragment, enabling systematic mapping of membrane protein topology.

One field where the PCA approach is especially powerful is for the detection of dually localized or ‘eclipsed’ proteins, where a portion of a protein population is found in small abundance at a specific location in the cell [79, 80]. To find such eclipsed proteins in the mitochondrial matrix, two complementary libraries were made. In the first, a C′‐α‐tag library [81] builds on the split‐β‐galactosidase assay [82, 83]. In this library, based on the C′‐SWAT collection, the small subunit of β‐galactosidase (termed α) was fused to the C′ of each protein to preserve the native gene promoter and MTS. Next, the library was mated with a strain expressing a matrix‐targeted omega fragment (the big subunit of β‐galactosidase). The resulting diploid library was then plated on Xgal, producing blue colonies if a protein entered or faced the mitochondrial matrix. Of course, it can also be used to assay dual localization to other cellular compartments or PPIs in general.

While the above strategy relies on an enzymatic reporter, which is a sensitive reporter for even a very small level of localization, it can only be assayed on galactose media. Hence, the second strategy was created to allow for a more versatile assay of the eclipsed proteome. This 3xGFP11 library [84] fuses three repeats of the small fragment of GFP to the C′ of proteins (building on the C′‐SWAT system). While this collection can be mated with any strain harboring the large subunit of GFP (1–10), to assay PPIs or co‐localization, it was utilized specifically to uncover mitochondrial matrix resident proteins by mating it to a strain expressing GFP1–10 from the mitochondrial genome. The presence of GFP1‐10 only in the matrix enabled specific detection of matrix‐localized or inner membrane proteins with matrix‐facing C′. It is important to note that split‐GFP systems are often regarded as irreversible. This limitation can be mitigated by careful experimental design or by using alternative, reversible PCA systems [85] when required. Together, these two collections revealed hundreds of potential dually localized ‘eclipsed’ proteins whose mitochondrial fraction was not visible by standard GFP libraries or PCA assays due to low mitochondrial abundance or dominant cytosolic pools.

Another SWAT‐based resource expanding the toolkit for interactome studies is the NanoBiT PPI libraries [86, 87]. NanoBiT is a reversible PCA using the Large Bit (LgBiT) and Small BiT (SmBiT) fragments of the NanoLuc luciferase [88]. Two genome‐wide libraries of opposite mating types were built on the C′‐SWAT system, one with LgBiT and the other with SmBiT, enabling systematic probing of PPIs in live cells using a ratiometric strategy. Complementing these, a genome‐wide full NanoLuc‐tagged library was also created to measure protein abundance and support NanoBiT data analysis.

For further details on these libraries, see Table 6.

**Table 6:** SWAT libraries for protein–protein interaction studies.

Library	Description	Location of tag	Mating type	Genotype	References	Coverage
N′ SWAT split DHFR	Derived from the parental N′‐SWAT collection, two libraries employ the split‐DHFR approach (See above) by seamless tagging. Mating of strains from opposite mating types is enabled by selection cassettes at a distal, inert locus	N′	MATa MATα	(a) *his3∆1 leu2∆0 met15∆0 ura3∆0 can1∆::GAL1pr‐SceI::STE2pr‐SpHIS5 lyp1∆::STE3pr‐LEU2 **chrV VCAJ1‐TPA1::NATR NATIVEpr‐ DHFR[1,2]‐XXX*** (α) *his3∆1 leu2∆0 met15∆0 ura3∆0 can1∆::GAL1pr‐SceI::STE2pr‐SpHIS5 lyp1∆::STE3pr‐LEU2 **chrV VCAJ1‐TPA1::HygroR NATIVEpr‐DHFR[3]‐XXX***	[38]	Genome‐wide
N′ SWAT split Venus	Derived from the parental N′‐SWAT collection, two libraries builds on the split‐Venus approach (See above)	N′	MATa MATα	(a) *his3∆1 leu2∆0 met15∆0 ura3∆0 can1∆::GAL1pr‐SceI::STE2pr‐SpHIS5 lyp1∆::STE3pr‐LEU2* ***G418R::CET1pr‐VN‐XXX*** (α) *his3∆1 leu2∆0 met15∆0 ura3∆0 can1∆::GAL1pr‐SceI::STE2pr‐SpHIS5 lyp1∆::STE3pr‐LEU2* ***HygroR‐TEF2pr‐VC‐XXX***	[38]	Genome‐wide
C′‐SWAT split‐β‐galactosidase	Based on the C′‐SWAT parental collection, each gene is altered such that the protein it encodes is fused to the alpha subunit of β‐galactosidase at its C terminus	C′	MATα	*his3∆1 leu2∆0 met15∆0 ura3∆0* *can1∆::GAL1pr‐SceI STE2pr‐spHIS5 lyp1∆::STE3pr‐LEU2* ***XXX‐alpha‐ADH1ter‐HygroR***	[81]	Genome‐wide
C′‐SWAT 3xGFP11	Based on the C′‐SWAT parental collection, each gene is altered such that the protein it encodes is C‐terminally tagged with three repeats of the small subunit of a split GFP (3×GFP11). The library also expresses MTS‐mCherry as a mitochondrial marker. The library can be mated with a strain harboring the big subunit of the split GFP (GFP1–10) for visualizing reconstitution of the complete GFP signal	C′	MATα	*his3∆1 leu2∆0 met15∆0 ura3∆0* *can1∆::GAL1pr‐SceI::STE2pr‐SpHIS5 lyp1∆::STE3pr‐LEU2* *ura3::NATR* *ho::MTS(Su9)‐mCherry‐MET15* ***XXX‐3xGFP11‐NATIVEter***	[84]	Genome‐wide
C′‐SWAT SmBiT	Based on the C′‐SWAT parental collection, A pair of libraries in which each gene is altered such that the protein it encodes is C‐terminally tagged with either SmBiT (Small BiT, MATa) or LgBiT (Large BiT, MATα) fragments of NanoLuc luciferase	C′	MATa	(a) *his3Δ1 leu2Δ0 met15Δ0 ura3Δ0* ***XXX‐SmBiT‐NATR** fcy1Δ::STE2pr‐spHIS5‐GAL1pr‐NLS‐SceI*	[86]	Genome‐wide
C′‐SWAT LgBiT			MATα	(α) *his3Δ1 leu2Δ0 met15Δ0 ura3Δ0* ***XXX‐LgBiT‐10HIS‐HygroR** can1Δ::STE3pr‐LEU2‐GAL1pr‐NLS‐SceI lyp1Δ*	[87]	
C′‐SWAT NanoLuc	Based on the C′‐SWAT parental collection, each gene is C‐terminally tagged with full‐length NanoLuc luciferase	C′	MATα	*his3Δ1 leu2Δ0 met15Δ0 ura3Δ0* ***XXX‐NanoLuc‐HygroR** can1Δ::STE3pr‐LEU2‐GAL1pr‐NLS‐SceI lyp1Δ*	[86]	Genome‐wide

The text that is not in bold indicates the genetic background of the library, and the text in bold indicates the genetic modification that was made to each and every gene in the library.

## Pushing the boundaries—next‐generation SWAT libraries

Building on the ease of library creation using the SWAT approach, several advanced libraries have recently been developed to tackle specific biochemical challenges.

While PPI mapping by PCAs and pull‐downs from strains expressing tagged proteins has been possible for many years, it relies on two fusion proteins (of the two split fragments) and mostly captures direct pairwise interactions. Recently, the development of proximity‐labeling tags has enabled more sensitive detection of PPIs, including weak and/or transient interactions, and allowed for neighborhood composition profiling. Leveraging this, a comprehensive suite of proximity‐labeling libraries was created using N′ fusions [89]. First, three libraries employ ‘promiscuous’ biotin ligases (which biotinylate any free lysine in their vicinity) fused to an HA tag. One collection uses the BioID2 enzyme [90], and two use the more active TurboID [91]. The HA tag enables classical co‐immunoprecipitation of proteins for the detection of stable interactors by western blot or MS, whereas transient interactors are detected via MS of biotinylated proteins following streptavidin‐mediated pull‐downs. Together, these dual tags help to distinguish between stable and transient protein associations. One of the TurboID‐HA collections also harbors the ABOLISH system—an auxin‐inducible strategy that transiently degrades yeast's endogenous biotin ligase (Bpl1) to reduce background biotinylation noise. ABOLISH improves the assay's sensitivity for detecting weak or fleeting interactions or those occurring between low‐abundance proteins.

In parallel, two ‘pairwise biotinylation’ libraries were constructed, built on the sequence‐specific *E. coli* biotin ligase, BirA, which only biotinylates an acceptor AVI peptide [92]. One library expresses the BirA ligase fused to each protein at its N′ (under their native promoters), and the other harbors a minimal AviTag peptide fused to each protein, also at the N′, in the opposite mating type. Both libraries have an ABOLISH background, and hence, the simple mating of a pair of proteins and the measurement of biotinylation by either western or a dot blot analysis (for higher throughput) [93] can allow for the measurement of the extent of interaction between them. Together, these proximity‐labeling collections greatly expand the ability to probe both stable complexes and transient associations on a proteome‐wide scale.

An important aspect of many yeast libraries is the capacity to isolate proteins with protein tags. However, the libraries described thus far that enable protein precipitation contain big tags. To enable protein detection with smaller tags, the C′‐SWAT Myc‐HRV (protease cleavage site)‐3XFlag tag library was created [94]. This allows for easy enrichment of the tagged proteins and purification of minimally perturbed proteins following cleavage. Moreover, it can even be used for organelle membrane purification.

C′ tag libraries do not enable the purification of proteins that are not adequately expressed by their native promoters under standard growth conditions. To enable widespread protein purification independent of promoter activity, an additional small tag library was created based on the N′‐SWAT collection. This collection introduced the *TEF1*pr‐3×HA tag into each gene [95]. Using the HA tag enables the sensitive detection of proteins, as with other small tags (e.g., by western‐ or dot blots), and allows for systematic *in vivo* analyses of protein abundance and post‐translational modifications. Furthermore, it can be used for localization studies when used in conjunction with the genetically encoded anti‐HA single‐chain variable fragment (scFV) [95, 96, 97] fused to a fluorophore.

One aspect of yeast collections that has been under‐explored is their use to measure local cellular conditions or accumulation of metabolites by fusing all proteins to the many available genetically encoded sensors that are constantly being developed. An example of the high potential of this approach is a collection that enables high‐resolution, local monitoring of hydrogen peroxide (H_2_O_2_) levels inside living yeast cells [98]. In this collection, the genetically encoded redox biosensor HyPer7 was fused to the C′ of proteins by utilizing the C′ SWAT collection. This allows detection of local redox environments with nanometer resolution. A control library using a redox‐insensitive mutant, SypHer7, was generated in parallel to control for redox‐independent effects.

To further understand how tag positioning affects protein function and fitness, N′‐SWAT and C′‐SWAT HaloTag libraries were developed [64]. Using the Halo‐3xMyc tag allowed direct comparison of fitness consequences associated with tag location. This systematic study underscored the importance of tag positioning, especially when considering protein functionality and localization. Moreover, many substrates have been developed for use with the Halo tag, including substrates compatible with super‐resolution microscopy, PPI profiling, purifications, protein‐DNA interaction measurements, and more [99], making them a valuable resource for using many methodologies in a high‐throughput manner.

These next‐generation libraries illustrate a trend toward highly specialized tagging strategies, each tailored to address a particular experimental need or limitation, from organelle‐centric proteolipid studies to mapping transient interactions or simply tagging with minimal footprints. This eruption of new tailor‐made libraries has been made possible due to the development of the SWAT method, allowing every laboratory to create its own library with ease.

For further details on these libraries, see Table 7.

**Table 7:** Next‐generation SWAT libraries.

Library	Description	Location of tag	Mating type	Genotype	References	Coverage
N′‐SWAT BioID‐HA tag	Derived from the parental N′‐SWAT collection, each gene is altered such that the protein it encodes is tagged at the N terminus with a BioID2‐HA tag under the control of the *CYC1* promoter. The BioID2 provides the capacity to biotinylate available lysine residues in proximal proteins	N′	MATα	*his3∆1 leu2∆0 met15∆0 ura3∆0* *can1∆::GAL1pr‐SceI‐STE2pr‐spHIS5 lyp1∆::STE3pr‐LEU2* ***HygroR‐CYC1pr‐BioID‐HA‐XXX***	[89]	Genome‐wide
N′‐SWAT BirA/ABOLISH	Derived from the parental N′‐SWAT collection, each gene is altered such that the protein it encodes is fused to BirA at its N terminus under its native promoters. BirA has the capacity to specifically biotinylated proteins carrying an AviTag. This library integrates the ABOLISH system, where the endogenous biotin ligase Bpl1 is fused to an AID* tag, allowing for controlled degradation of Bpl1 to reduce background biotinylation	N′	MATa	*his3∆1 leu2∆0 met15∆0 ura3∆0* *can1∆::GAL1pr‐SceI‐STE2pr‐spHIS5 lyp1∆::STE3pr‐LEU2* BPL1‐AID*‐9myc‐NATR Nativepr‐BirA‐XXX	[89]	Genome‐wide
N′‐SWAT AviTag/ABOLISH	Derived from the parental N′‐SWAT collection, each gene is altered such that the protein it encodes has an N‐terminal AviTag fusions seamlessly integrated, allowing native promoter regulation on the background of the OsTir1 adaptor. This library should be used in combination with the BirA library, and they are created in opposite mating types. The library also incorporates the ABOLISH system	N′	MATα	*leu2∆0 met15∆0 ura3∆0* *can1∆::GAL1pr‐SceI‐STE2pr‐spHIS5 lyp1∆::STE3pr‐LEU2* BPL1‐AID*‐6HA‐HygroR his3∆1::OsTIR1‐HIS3 Nativepr‐AviTag‐XXX	[89]	Genome‐wide
N′‐SWAT TurboID‐HA tag	Derived from the parental N′‐SWAT collection, each gene is altered such that the protein it encodes is tagged at the N terminus with TurboID‐HA under the control of the *CYC1* promoter. TurboID is a highly active biotin ligase capable of rapidly biotinylating proximal proteins on available lysine residues	N′	MATα	*his∆1 leu2∆0 met15∆0 ura3∆0* *can1∆::GAL1pr‐SceI‐STE2pr‐spHIS5 lyp1∆::STE3pr‐LEU2* ***HygroR‐CYC1pr‐TurboID‐HA‐XXX***	[89]	Genome‐wide
N′‐SWAT TurboID‐HA tag/ABOLISH	Derived from the parental N′‐SWAT collection, this library enhances the TurboID‐HA N′ library by incorporating the ABOLISH system	N′	MATα	*leu2∆0 met15∆0 ura3∆0* *can1∆::GAL1pr‐SceI‐STE2pr‐spHIS5 lyp1∆::STE3pr‐LEU2* HygroR‐CYC1pr‐TurboID‐HA‐XXX, BPL1‐AID*‐9myc‐G418R his3∆1::OsTIR1‐HIS3	[89]	Genome‐wide
C′‐SWAT Myc‐HRV‐Flag tag	Based on the C′‐SWAT parental collection, each gene is altered such that the protein it encodes is tagged with a C‐terminal myc epitope followed by a human rhinovirus (HRV) 3C protease cleavage site, and a 3xFLAG tag for efficient protein purification	C′	MATa	*his3∆1 leu2∆0 met15∆0 ura3∆0* *can1∆::GAL1pr‐SceI‐NLS‐STE2pr‐spHIS5 lyp1∆::STE3pr‐LEU2* ***XXX‐myc‐HRV‐3xFlag‐ADH1term‐G418R***	[94]	Genome‐wide
N′‐SWAT HaloTag	Derived from the parental N′ and C′‐SWAT collections, each gene is altered such that the protein it encodes is fused with the Halo tag, either at its N‐ or C terminus (respectively)	N′	MATα	*his3Δ1 ura3Δ0 met15Δ0* *lyp1Δ can1Δ::STE3pr‐LEU2‐GAL1pr‐NLS‐I‐SCEI* ***leu2Δ::NATR‐TEF1pr‐mNeonGreen‐CYC1term pdr5Δ::HygroR*** ***NATIVEpr‐HaloTag‐3myc‐XXX***	[64]	Genome‐wide
C′‐SWAT HaloTag		C′	MATα	*his3Δ1 ura3Δ0 met15Δ0* *lyp1Δ can1Δ::STE3pr‐LEU2‐GAL1pr‐NLS‐I‐SCEI* ***leu2Δ::NATR‐TEF1pr‐mCherry‐CYC1term pdr5Δ::HygroR*** ***XXX‐HaloTag‐3myc‐NATIVEterm***	[64]	
C′‐SWAT H_2_O_2_ biosensor	Based on the C′‐SWAT parental collection, each gene is altered such that the protein it encodes is C‐terminally tagged with the hydrogen peroxide (H_2_O_2_) sensor HyPer7, enabling the detection of local redox changes at the level of individual proteins. A corresponding control library using a redox‐insensitive mutant of the sensor (SypHer7) allows to detect and exclude nonspecific sensor responses	C′	MATa	*his3Δ1 ura3Δ0 leu2Δ0::GAL1pr‐NLS‐I‐SCEI‐natNT2can1Δ::STE2pr‐SpHIS5 lyp1Δ::STE3pr‐LEU2 **XXX‐HyPer7‐ADH1term‐HygroR or XXX‐SypHer7‐ADH1term‐HygroR***	[98]	Genome‐wide
N′‐SWAT HA tag	Derived from the parental N′‐SWAT collection, each gene is altered such that the protein it encodes is N‐terminally tagged with a 3×HA epitope under the regulation of the TEF1 promoter	N′	MATa	*his3∆1 leu2∆0 met15∆0 ura3∆0 **TEF1pr‐3HA‐XXX‐NATR*** *can1∆::GAL1pr‐SceI‐STE2pr‐spHIS5 lyp1∆::STE3pr‐LEU2*	[95]	Genome‐wide

The text that is not in bold indicates the genetic background of the library, and the text in bold indicates the genetic modification that was made to each and every gene in the library.

## Beyond the horizon—the future of yeast libraries

Unlike plasmid or CRISPR‐based systems in higher eukaryotes, yeast libraries offer fully integrated, genome‐wide, stable genomic modifications at scale, with unparalleled ease of use, reproducibility, and genetic tractability—advantages that continue to make them uniquely powerful even in the age of high‐throughput CRISPR screens. To this end, there is much room to expand on the utility of yeast collections.

The past two decades have demonstrated how systematically engineered yeast libraries can redefine the study of biology, from mapping gene function and protein localization to dissecting interaction networks and cellular organization. As these resources mature, the next generation of yeast libraries is expected to move beyond static perturbations toward fully dynamic, programmable, and multiplexed systems. With the constant development of new tags and methodologies, the design principles will shift from one‐size‐fits‐all collections to modular, condition‐responsive libraries tailored to specific biological or technological questions.

Recently, the fluorescent protein (FP) field has expanded rapidly, with over 450 new FPs and variants developed in the past decade [100]. These include smaller, brighter proteins and probes optimized for advanced techniques, such as energy transfer, time‐resolved imaging, light‐induced protein activation, super‐resolution microscopy, molecular sensors, and new self‐labeling tags. However, most current fluorescent yeast libraries rely on early‐generation FPs, which are often incompatible with modern imaging approaches and limit the resolution of discoveries made by them.

Given recent advances in probe development, there is a timely opportunity to revisit the design of fluorescent yeast libraries. Incorporating more advanced, spectrally diverse, and photophysically robust fluorophores could expand the range of biological questions addressable in yeast and provide a foundation for future studies of dynamic, high‐resolution proteome behavior.

Nonetheless, existing libraries can also be repurposed beyond their original goals with newly developed methods. For example, the original C′‐GFP library, initially developed for fluorescence‐based localization, was recently used to map the yeast protein interactome using high‐throughput affinity enrichment MS [101]. This work resulted in a high‐confidence interaction network of over 30 000 interactions and exemplifies how stable, genomically integrated libraries can be leveraged for diverse, large‐scale biochemical and systems‐level studies.

Moreover, machine learning and artificial intelligence (AI) advances will match the expanding scale and complexity of current and future libraries. As evident from datasets from imaging, transcriptomics, proteomics, and genetic/physical interaction of existing libraries become richer and more multidimensional [31, 34, 35, 37], AI will most certainly play an increasing role in extracting patterns, predicting gene functions, and identifying previously unrecognized or subtle functional relationships between components. Future libraries will not only be tools for experimentation but also training grounds for predictive models of cellular behavior. We can anticipate a feedback loop where AI guides the design of libraries that can overcome the limitations of past libraries, and libraries, in turn, generate the data to refine AI‐driven insights.

Ultimately, we expect future yeast libraries to be more adaptive, interpretable, and interconnected, serving as living platforms that bridge systematic experimentation with computational discovery. In this vision, yeast remains not just a simple model organism but a blueprint for engineering and understanding complex biological systems at scale, ultimately enabling the creation of the first model of a fully functional fundamental eukaryotic cell.

## Conflict of interest

The authors declare that they have no competing interests.

## Author contributions

DB contributed to the data curation and visualization. MS contributed to the funding acquisition. OK and MS contributed to the supervision. All authors contributed to the conceptualization, writing—original draft, and writing—review and editing.
